# A nomogram model for predicting the risk of checkpoint inhibitor‐related pneumonitis for patients with advanced non‐small‐cell lung cancer

**DOI:** 10.1002/cam4.6244

**Published:** 2023-07-06

**Authors:** Yao Zhang, Lincheng Zhang, Shuhui Cao, Yue Wang, Xuxinyi Ling, Yan Zhou, Hua Zhong

**Affiliations:** ^1^ Department of Respiratory and Critical Care Medicine Shanghai Chest Hospital, Shanghai Jiao Tong University School of Medicine Shanghai China

**Keywords:** checkpoint inhibitor‐related pneumonitis (CIP), immunotherapy, nomogram, non‐small‐cell lung cancer (NSCLC), predict

## Abstract

**Objective:**

Immunotherapy extensively treats advanced non‐small‐cell lung cancer (NSCLC). Although immunotherapy is generally better tolerated than chemotherapy, it can cause multiple immune‐related adverse events (irAEs) involving multiple organs. Checkpoint inhibitor‐related pneumonitis (CIP) is a relatively uncommon irAE that, in severe cases, can be fatal. Potential risk factors for the occurrence of CIP are currently poorly understood. This study sought to develop a novel scoring system for predicting the risk of CIP based on a nomogram model.

**Methods:**

We retrospectively collected advanced NSCLC patients who received immunotherapy at our institution between January 1, 2018, and December 30, 2021. All patients who met the criteria were randomly divided into the training set and testing set (in a ratio of 7:3), and cases fulfilling the CIP diagnostic criteria were screened. The patients' baseline clinical characteristics, laboratory tests, imaging, and treatment information were extracted from the electronic medical records. The risk factors associated with the occurrence of CIP were identified based on the results of logistic regression analysis on the training set, and a nomogram prediction model was developed. The discrimination and prediction accuracy of the model was evaluated using the receiver operating characteristic (ROC) curve, the concordance index (C‐index), and the calibration curve. Decision curve analysis (DCA) was used to evaluate the clinical applicability of the model.

**Results:**

The training set comprised 526 (CIP: 42 cases), and the testing set comprised 226 (CIP: 18 cases) patients, respectively. In the training set, the final multivariate regression analysis revealed that age (*p* = 0.014; odds ratio [OR] = 1.056; 95% Confidence Interval [CI] =1.011–1.102), Eastern Cooperative Oncology Group performance status (*p* = 0.002; OR = 6.170; 95% CI = 1.943–19.590), history of prior radiotherapy (*p* < 0.001; OR = 4.005; 95% CI = 1.920–8.355), baseline white blood cell count (WBC) (*p* < 0.001; OR = 1.604; 95% CI = 1.250–2.059), and baseline absolute lymphocyte count (ALC) (*p* = 0.034; OR = 0.288; 95% CI = 0.091–0.909) were identified as independent risk factors for the occurrence of CIP. A prediction nomogram model was developed based on these five parameters. The area under the ROC curve and C‐index of the prediction model in the training set and testing set were 0.787 (95% CI: 0.716–0.857) and 0.874 (95% CI: 0.792–0.957), respectively. The calibration curves are in good agreement. The DCA curves indicate that the model has good clinical utility.

**Conclusion:**

We developed a nomogram model that proved to be a good assistant tool for predicting the risk of CIP in advanced NSCLC. This model has the potential power to help clinicians in making treatment decisions.

## 1 INTRODUCTION

Lung cancer is the primary cause of cancer‐related mortality worldwide.^1^ Non‐small‐cell lung cancer (NSCLC) is the most prevalent pathological subtype of lung cancer, accounting for 80%–85% of all cases. At present, immune checkpoint inhibitors (ICIs), such as anti‐programmed cell death protein‐1 (PD‐1) and anti‐programmed cell death ligand‐1 (PD‐L1) agents, have become one of the standard therapies for advanced NSCLC.^2^ These agents exhibit durable treatment responses in patients without treatable driver mutations, particularly those with higher PD‐L1 expression. Moreover, immunotherapy is more well‐tolerated for patients than conventional treatments such as systemic radiotherapy and chemotherapy.

However, immunotherapy also causes a broad spectrum of immune‐related adverse events (irAEs) that can affect multiple organ system.^3^ Previous clinical study has indicated that checkpoint inhibitor‐related pneumonitis (CIP) is a relatively uncommon AE in patients with advanced lung cancer who received ICIs therapy combination or monotherapy.^4^ CIP refers to the rapid development of respiratory symptoms (such as cough, dyspnea, polypnea, and other signs of hypoxia) and new pulmonary infiltrates on chest imaging. Meanwhile, pulmonary infection and malignant lung infiltration must be ruled out.^5^ Compared to patients with other types of cancer, NSCLC patients receiving immunotherapy have a higher risk of developing CIP.^6^ According to clinical trial research, the incidence of all‐grade CIP ranges from 1.4% to 8.5%, and the incidence of grade 3–4 CIP ranges from 1.0% to 3.4%.^7^ However, the incidence of CIP tends to be higher in the real world.

CIP may lead to severe clinical outcomes, which tend to be overlooked by clinicians due to the overall low incidence of irAEs. The standard treatment for ≥Grade 3 CIP remains high doses of corticosteroid or combined with immunosuppressive agents if necessary.^8^ However, clinical responses may be limited due to delayed diagnosis or hesitation to increase steroid dosage, resulting in irreversible harm to patients' pulmonary function or even life‐threatening circumstances.^9^ In fact, a multicenter retrospective investigation discovered that most patients diagnosed with CIP were high‐severity cases, indicating that symptoms usually will not relieve with simple cessation of ICI therapy in CIP patients.^10^ The study focused on 30 patients diagnosed with CIP among 315 ICI‐treated lung cancer patients, of whom 73.3% presented with low oxygen saturation (<88%) at diagnosis, 93.3% were treated by corticosteroids, and 27% died during the treatment of CIP.^10^ Another real‐world retrospective trial reported that although overall survival was similar between CIP and non‐CIP NSCLC patients who received ICIs, the risk of mortality in patients with grade ≥2 CIP increased several folds (HR 2.54, 95% CI 1.20–5.34, *p* = 0.014).^11^

Therefore, it is crucial to prevent and monitor the occurrence of CIP in advanced NSCLC patients receiving ICIs. Since CIP occurs in a relatively small population, it is essential to identify patients with risk factors for CIP. Previous studies demonstrated that a history of chronic pulmonary disease, prior thoracic radiotherapy, and abnormality in some peripheral‐blood parameters are associated with occurrences of CIP.^4^, ^10^, ^11^ Cho et al.^7^ revealed that NSCLC patients with interstitial lung disease have a significantly higher incidence of CIP when receiving ICIs (OR = 6.03, 95% CI 1.19–30.45, *p* = 0.030). In terms of peripheral blood inflammatory biomarkers, previous study confirmed that increased baseline peripheral blood absolute eosinophil count (AEC ≥ 0.125 × 10^9^ cells/L) levels were associated with an increased risk of CIP (*p* < 0.001).^12^, ^13^

However, researches are scarce regarding establishing a CIP risk prediction model, which might be helpful in practice for clinicians to rule out a small portion of patients at significant risk of developing CIP. Recently, nomogram has been widely used in oncology to predict the efficacy and safety of cancer therapy as it spares clinicians from complicated calculations used in traditional risk prediction models. Multiple studies on lung cancer patients receiving immunotherapy have employed nomograms to predict overall survival probabilities based on clinicopathologic features.^14^, ^15^ Therefore, in this study, we have conducted a nomogram prediction model through comprehensive univariate and multivariate analysis of risk factors associated with the occurrence of CIP. This model can provide clinicians with a rapid and intuitive tool to assess the probability of CIP in NSCLC patients treated with ICI based on typical clinical and laboratory characteristics, thereby assessing patients at an early stage for prevention and monitoring throughout treatment, assisting clinicians in making a treatment decision.

## 2 METHODS

### 2.1 Patients

This retrospective study selected patients pathologically diagnosed with advanced NSCLC and treated with ICIs at our institution between January 1, 2018, and December 30, 2021. Patients were enrolled according to the following criteria: (a) All patients were pathologically diagnosed with NSCLC according to the World Health Organization histological classification; (b) patients with stage IIIB‐IV NSCLC according to the eighth edition of the tumor‐ nodal‐ metastasis staging system; (c) patients with complete clinical and imaging data for evaluation are available; (d) Eastern Cooperative Oncology Group performance status (ECOG PS): 0–2; and (e) age ≥18 years old. We excluded patients with pre‐existing radiation‐associated pneumonia or other severe interstitial pneumonia and lung infections before treatment with ICIs and patients who underwent radiotherapy after receiving ICIs and developing pneumonitis. All eligible patients were randomly divided into a training and testing set at a 7:3 ratio. This study has been approved by the Ethics Committee of our institution (No. KS(Y)21310) and was conducted following the Declaration of Helsinki (as revised in 2013).

### 2.2 Diagnosis of CIP

CIP is defined as the development of rapidly progressive respiratory symptoms in patients treated with ICIs (for example, cough, dyspnea, fever, and chest pain) with new pulmonary infiltrates on chest imaging and exclusion of other lung diseases such as pulmonary infection and tumor progression.^5^ There is no unified standard for the diagnosis of CIP, and the final diagnosis is primarily determined by ruling out related diseases. The diagnosis of CIP in this study is based on the 2019 NCCN guidelines for the management of immunotherapy‐related toxicities.^16^ Two experienced respiratory oncologists and radiologists conducted the final clinical diagnosis of the patient through a comprehensive review and analysis of the patient's clinical symptoms, laboratory testing data, and radiographic findings, meanwhile entirely excluding the other pulmonary disease (for example, pulmonary infection, tumor progression, and radiation pneumonitis). Patients were not classified as CIP if they could not be distinguished as radiation‐associated pneumonia or CIP. The CIP was graded according to the patient's clinical symptoms and the extent of pneumonia on chest CT.^16^

### 2.3 Data collection

The data were extracted entirely from electronic medical records. We have collected patients' demographics, clinical and treatment information, including gender, age, smoking history, ECOG PS, previous diabetes history, previous radiotherapy, tumor staging, tumor histological type, molecular mutation, PD‐L1 status, metastatic sites, treatment lines, the type of combination therapy, and type of ICI. The peripheral blood laboratory data of all patients were collected before the initiation of ICI treatment, including white blood cell count (WBC) (×10^9^/L), absolute neutrophil count (ANC) (×10^9^/L), ALC (×10^9^/L), AEC (×10^9^/L), neutrophil‐to‐lymphocyte ratio (NLR), lactate dehydrogenase (LDH) (U/L), and C‐reactive protein (CRP) (mg/L).

### 2.4 Statistical analysis

Continuous parameters are presented as the median (range), and Mann—Whitney *U*‐test is used to compare CIP and non‐CIP groups. The categorical variables are presented as the number and percentage, and the chi‐squared test or Fisher's exact test is used to compare categorical variables between two groups. A two‐sided *p* < 0.05 is considered significant. The correlates of CIP predisposition are evaluated by univariate logistic regression analysis, and the variables with *p* < 0.05 are entered into the multivariate analysis. The independent risk factors are determined based on the multivariate analysis and are selected to build a nomogram model. The concordance index (C‐index), receiver operating characteristic (ROC) curves, and calibration curves were used to qualify the discrimination and prediction performance of the nomogram model. The decision curve analysis (DCA) was used to evaluate the clinical utility of the model. The C‐index value higher than 0.70 was thought to indicate a better degree of discrimination, and 1000 bootstrap resamplings were performed to validate this model.

Statistical analyses and graphics are performed using the SPSS (SPSS statistical software for Windows, version 24; IBM) and R v. 3.6.3 software (The R Foundation for Statistical Computing).

## 3 RESULTS

### 3.1 Patients characteristics

**FIGURE 1:**
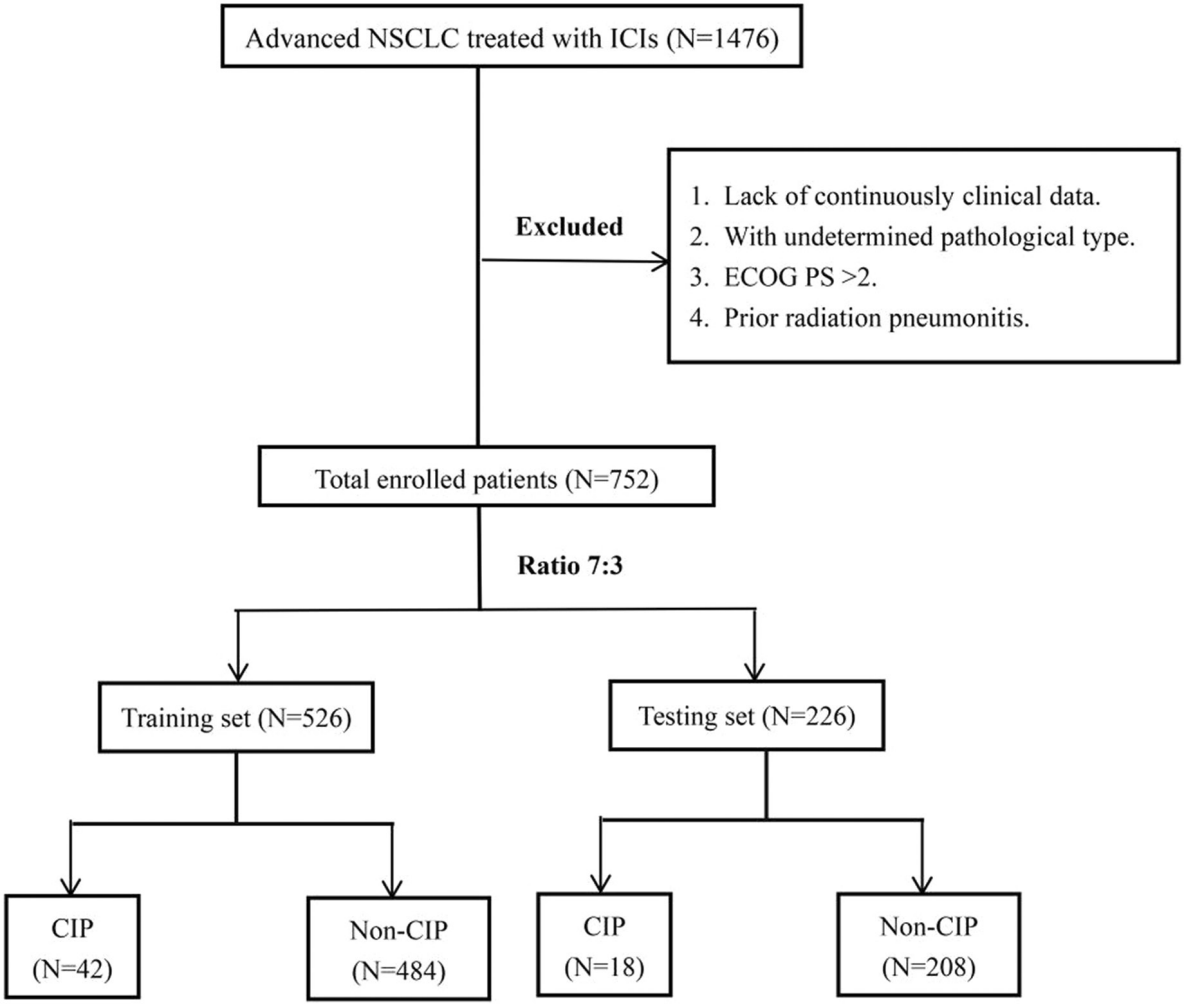
Flowchart of patient selection.

This study ultimately included 752 patients with advanced NSCLC treated with ICIs between January 1, 2018, and December 30, 2021, of whom 60 were diagnosed with CIP, for an incidence of 8.0% (60/752). All patients were divided into the training set and testing set in a 7:3 ratio (526 patients and 226 patients, respectively). Finally, 42 patients in the training set were confirmed to have CIP, while 18 patients in the testing set were diagnosed. The procedure for patient selection is shown in Figure 1.

The baseline characteristics of patients in the training set and testing set are shown in Table 1. In both the training and testing set, the preponderance of patients was men under 70 years old. Only age (*p* = 0.024 and *p* = 0.040, respectively) and ECOG PS (*p* < 0.001 for both the training and testing set) showed significant differences between CIP and Non‐CIP groups. Notably, more patients with CIP had a history of radiotherapy compared with the Non‐CIP group (*p* < 0.001 for both the training and testing set). Besides, patients with baseline higher WBC (10^9^/L), baseline higher NLR, and baseline lower ALC (10^9^/L) were more likely to suffer CIP in both the training set and testing set (WBC: *p* = 0.011 and 0.024; NLR: *p* = 0.029 and 0.003; ALC: *p* = 0.035 and 0.004, respectively). Other baseline clinical characteristics, treatment information, and laboratory data showed no difference between the CIP and non‐CIP groups in the training and testing set.

**TABLE 1:** Baseline characteristics of patients in training and testing set.

Variables	Training set			Testing set		
	CIP (*N* = 42)	Non‐CIP (*N* = 484)	*p* value	CIP (*N* = 18)	Non‐CIP (*N* = 208)	*p* value
Age, years	66 (46–83)	63 (21–86)	0.024	68 (59–82)	64 (32–84)	0.040
Median (range)						
≥70 years, *n* (%)	11 (26.2)	96 (19.8)	0.326	8 (44.4)	50 (24.0)	0.057
<70 years, *n* (%)	31 (73.8)	388 (80.2)		10 (55.6)	158 (76.0)	
Gender, *n* (%)			0.201			0.225
Male	37 (88.1)	387 (80.0)		17 (94.4)	174 (83.7)	
Female	5 (11.9)	97 (20.0)		1 (5.6)	34 (16.3)	
TNM stage, *n* (%)			0.099			0.075
IIIB‐IIIC	7 (16.7)	43 (8.9)		3 (16.7)	12 (5.8)	
IV	35 (83.3)	441 (91.1)		15 (83.3)	196 (94.2)	
ECOG PS, *n* (%)			<0.001			<0.001
0–1	36 (85.7)	473 (97.7)		13 (72.2)	203 (97.6)	
2	6 (14.3)	11 (2.3)		5 (27.8)	5 (2.4)	
Smoking history, *n* (%)			0.465			0.754
Current/former	32 (76.2)	343 (70.9)		12 (66.7)	146 (70.2)	
Never	10 (23.8)	141 (29.1)		6 (33.3)	62 (29.8)	
Diabetes, *n* (%)			0.448			0.125
Yes	3 (7.1)	22 (4.5)		3 (16.7)	14 (6.7)	
No	39 (92.9)	462 (95.5)		15 (83.3)	194 (93.3)	
Pre‐existing interstitial lung disease, *n* (%)			0.586			0.367
Yes	4 (9.5)	35 (7.2)		2 (11.1)	12 (5.8)	
No	38 (90.5)	449 (92.8)		16 (88.9)	196 (94.2)	
Pre‐existing lung emphysema, *n* (%)			0.235			0.566
Yes	8 (19.0)	61 (12.6)		3 (16.7)	25 (12.0)	
No	34 (81.0)	423 (87.4)		15 (83.3)	183 (88.0)	
Histologic type, *n* (%)			0.151			0.573
Adenocarcinoma	19 (45.2)	286 (59.1)		9 (50.0)	121 (58.2)	
Squamous	15 (35.7)	144 (29.8)		8 (44.4)	68 (32.7)	
Other NSCLC	8 (19.0)	54 (11.2)		1 (5.6)	19 (9.1)	
PD‐L1 TPS, *n* (%)			0.434			0.903
<1%	12 (28.6)	105 (21.7)		6 (33.3)	59 (28.4)	
≥1%	11 (26.2)	168 (34.7)		6 (33.3)	73 (35.1)	
Unknown	19 (45.2)	211 (43.6)		6 (33.3)	76 (36.5)	
Molecular mutation, *n* (%)			0.341			0.891
EGFR/ALK	2 (4.8)	44 (9.1)		2 (11.1)	21 (10.1)	
Wild type	40 (95.2)	440 (90.9)		16 (88.9)	187 (89.9)	
Brain metastasis, *n* (%)			0.235			0.560
Yes	4 (9.5)	80 (16.5)		2 (11.1)	34 (16.3)	
No	38 (90.5)	404 (83.5)		16 (88.9)	174 (83.7)	
No. of metastasis site, *n* (%)			0.075			0.112
0–1	10 (23.8)	182 (37.6)		3 (16.7)	73 (35.1)	
≥2	32 (76.2)	302 (62.4)		15 (83.3)	135 (64.9)	
Therapy lines of ICI, *n* (%)			0.379			0.120
1	18 (42.9)	181 (37.4)		6 (33.3)	92 (44.2)	
2	17 (40.5)	175 (36.2)		10 (55.6)	67 (32.2)	
≥3	7 (16.7)	128 (26.4)		2 (11.1)	49 (23.6)	
Combination with chemotherapy, *n* (%)			0.072			0.554
Yes	27 (64.3)	241 (49.8)		9 (50.0)	119 (57.2)	
No	15 (35.7)	243 (50.2)		9 (50.0)	89 (42.8)	
Combination with anti‐angiogenesis therapy, *n* (%)			0.093			0.149
Yes	3 (7.1)	83 (17.1)		1 (5.6)	40 (19.2)	
No	39 (92.9)	401 (82.9)		17 (94.4)	168 (80.8)	
Prior radiotherapy, *n* (%)			<0.001			<0.001
Yes	17 (40.5)	85 (17.6)		9 (50.0)	31 (14.9)	
No	25 (59.5)	399 (82.4)		9 (50.0)	177 (85.1)	
Type of ICI, *n* (%)			0.060			0.221
Anti‐PD‐1	39 (92.9)	473 (97.7)		17 (94.4)	206 (99.0)	
Anti‐PD‐L1	3 (7.1)	11 (2.3)		1 (5.6)	2 (1.0)	
Peripheral blood biomarkers						
Median (range)						
WBC (×10^9^/L)	7.8 (3.7–16.4)	6.9 (2.9–11.2)	0.011	7.9 (6.1–27.8)	7.1 (2.0–11.0)	0.024
ANC (×10^9^/L)	5.0 (0.5–10.6)	4.6 (0.6–9.8)	0.324	4.8 (3.3–9.3)	4.6 (1.4–8.6)	0.467
ALC (×10^9^/L)	1.4 (0.3–2.9)	1.5 (0.3–4.6)	0.035	1.2 (0.4–2.1)	1.5 (0.4–4.5)	0.004
AEC (×10^9^/L)	0.2 (0.0–0.6)	0.1 (0.0–1.5)	0.774	0.1 (0.0–0.7)	0.1 (0.0–1.2)	0.626
NLR	4.2 (1.5–16.3)	3.1 (0.1–23.7)	0.029	4.6 (1.7–13.3)	3.1 (0.6–19.3)	0.003
LDH (U/L)	201.5 (133.0–587.0)	200.0 (125.0–732.0)	0.620	242.0 (133.0–369.0)	205.5 (111.0–756.0)	0.088
CRP (mg/L)	10.4 (0.8–164.5)	5.4 (0.1–153.6)	0.132	15.4 (0.5–234.4)	5.3 (0.3–132.0)	0.362

Abbreviations: AEC, absolute eosinophil count; ALC, absolute lymphocyte count; ALK, anaplastic lymphoma kinase; ANC, absolute neutrophil count; CIP, checkpoint inhibitor‐related pneumonitis; CRP, C‐reactive protein; ECOG PS, Eastern Cooperative Oncology Group, performance status; EGFR, epidermal growth factor receptor; ICI, immune checkpoint inhibitor; LDH, lactate dehydrogenase; NLR, neutrophil to lymphocyte ratio; NSCLC, non‐small cell lung cancer; PD‐1, programmed cell death protein‐1; PD‐L1, programmed cell death ligand‐1; TNM, tumor node metastasis; TPS, tumor proportion score; WBC, white blood cell count.

### 3.2 Univariate and multivariate analysis of risk factors associated with CIP

Results of univariate and multivariate analysis in the training set are shown in Table 2. According to the univariate analysis, the age, ECOG PS, history of prior radiotherapy, WBC, ALC, and NLR are all significant differences between the CIP and non‐CIP groups (*p* < 0.05). Afterward, these six variables were chosen for multivariate analysis. The subsequent multivariate analysis in the training set reveals that the age (*p* = 0.014; OR = 1.056; 95% CI = 1.011–1.102), ECOG PS (*p* = 0.002; OR = 6.170; 95% CI = 1.943–19.590), history of prior radiotherapy (*p* < 0.001; OR = 4.005; 95% CI = 1.920–8.355), WBC (*p* < 0.001; OR = 1.604; 95% CI = 1.250–2.059), and ALC (*p* = 0.034; OR = 0.288; 95% CI = 0.091–0.909) are strong predictors of CIP occurrence. These five variables were eventually identified as independent predictive factors that indicated CIP and were selected to build a predictive nomogram model.

**TABLE 2:** Univariate and multivariate logistic analysis for risk factors of CIP in training set.

Variables	Univariate analysis		Multivariate analysis	
	OR (95% CI)	*p* value	OR (95% CI)	*p* value
Age	1.053 (1.012–1.095)	0.010	1.056 (1.011–1.102)	0.014
TNM stage (IV vs. IIIB–IIIC)	0.488 (0.204–1.163)	0.105	–	–
ECOG PS (2 vs. 0–1)	7.167 (2.506–20.497)	<0.001	6.170 (1.943–19.590)	0.002
Therapy line (1st–2nd vs. ≥3rd)	1.798 (0.779–4.149)	0.169	–	–
Combination with chemotherapy (Yes vs. No)	1.815 (0.942–3.497)	0.075	–	–
Combination with anti‐angiogenesis therapy (Yes vs. No)	0.372 (0.112–1.231)	0.105	–	–
Prior radiotherapy (Yes vs. No)	3.192 (1.651–6.170)	0.001	4.005 (1.920–8.355)	<0.001
WBC (10^9^/L)	1.320 (1.116–1.561)	0.001	1.604 (1.250–2.059)	<0.001
ALC (10^9^/L)	0.483 (0.260–0.898)	0.021	0.288 (0.091–0.909)	0.034
NLR	1.143 (1.027–1.272)	0.014	0.848 (0.653–1.102)	0.217

Abbreviations: ALC, absolute lymphocyte count; CIP, checkpoint inhibitor‐related pneumonitis; ECOG PS, Eastern Cooperative Oncology Group, performance status; NLR, neutrophil to lymphocyte ratio; TNM, tumor node metastasis; WBC, white blood cell count.

Similarly, the univariate and multivariate analysis of the testing set demonstrate that age, ECOG PS, history of prior radiotherapy, WBC, and ALC are potent predictors of the occurrence of CIP (*p* < 0.05) (Table 3).

**TABLE 3:** Univariate and multivariate logistic analysis for risk factors of CIP in testing set.

Variables	Univariate analysis		Multivariate analysis	
	OR (95% CI)	*p* value	OR (95% CI)	*p* value
Age	1.070 (1.008–1.137)	0.027	1.084 (1.001–1.175)	0.047
TNM stage (IV vs. IIIB–IIIC)	0.306 (0.078–1.204)	0.090	–	–
ECOG PS (2 vs. 0–1)	15.615 (4.006–60.872)	<0.001	26.018 (3.328–203.385)	0.002
Therapy line (1st–2nd vs. ≥3rd)	2.465 (0.548–11.099)	0.240	–	–
Combination with chemotherapy (Yes vs. No)	0.748 (0.285–1.961)	0.555	–	–
Combination with anti‐angiogenesis therapy (Yes vs. No)	0.247 (0.032–1.912)	0.180	–	–
Prior radiotherapy (Yes vs. No)	5.710 (2.101–15.515)	0.001	6.548 (1.755–24.431)	0.005
WBC (10^9^/L)	1.290 (1.024–1.626)	0.031	2.062 (1.187–3.583)	0.010
ALC (10^9^/L)	0.214 (0.074–0.617)	0.004	0.025 (0.001–0.428)	0.011
NLR	1.271 (1.092–1.480)	0.002	0.663 (0.390–1.125)	0.128

Abbreviations: ALC, absolute lymphocyte count; CIP, checkpoint inhibitor‐related pneumonitis; ECOG PS, Eastern Cooperative Oncology Group, performance status; NLR, neutrophil to lymphocyte ratio; TNM, tumor nodal metastasis; WBC, white blood cell count.

### 3.3 Nomogram model establishment and validation

**FIGURE 2:**
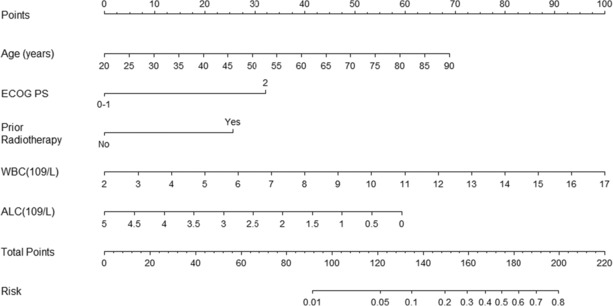
Nomogram model for predicting the occurrence of checkpoint inhibitor‐related pneumonitis (CIP) in advanced non‐small‐cell lung cancer (NSCLC) patients receiving immune checkpoint inhibitors (ICIs) treatment.

By using ROC curves to determine the optimal thresholds for these factors, we identified that the age (>61 years), history of radiotherapy, ECOG PS score of 2, higher baseline WBC (>7.1 × 10^9^/L), and lower baseline ALC (<1.0 × 10^9^/L) as independent risk factors for the occurrence of CIP. Based on these five risk factors, a nomogram predictive model was developed (Figure 2). In the nomogram model, each factor corresponds to a score at the top (Points). A total score (total points) can be obtained by adding up the scores for each factor. Based on this total score, the predicted risk value (risk) can be found in the last row, representing the proportion of risk for a patient developing CIP.

**FIGURE 3:**
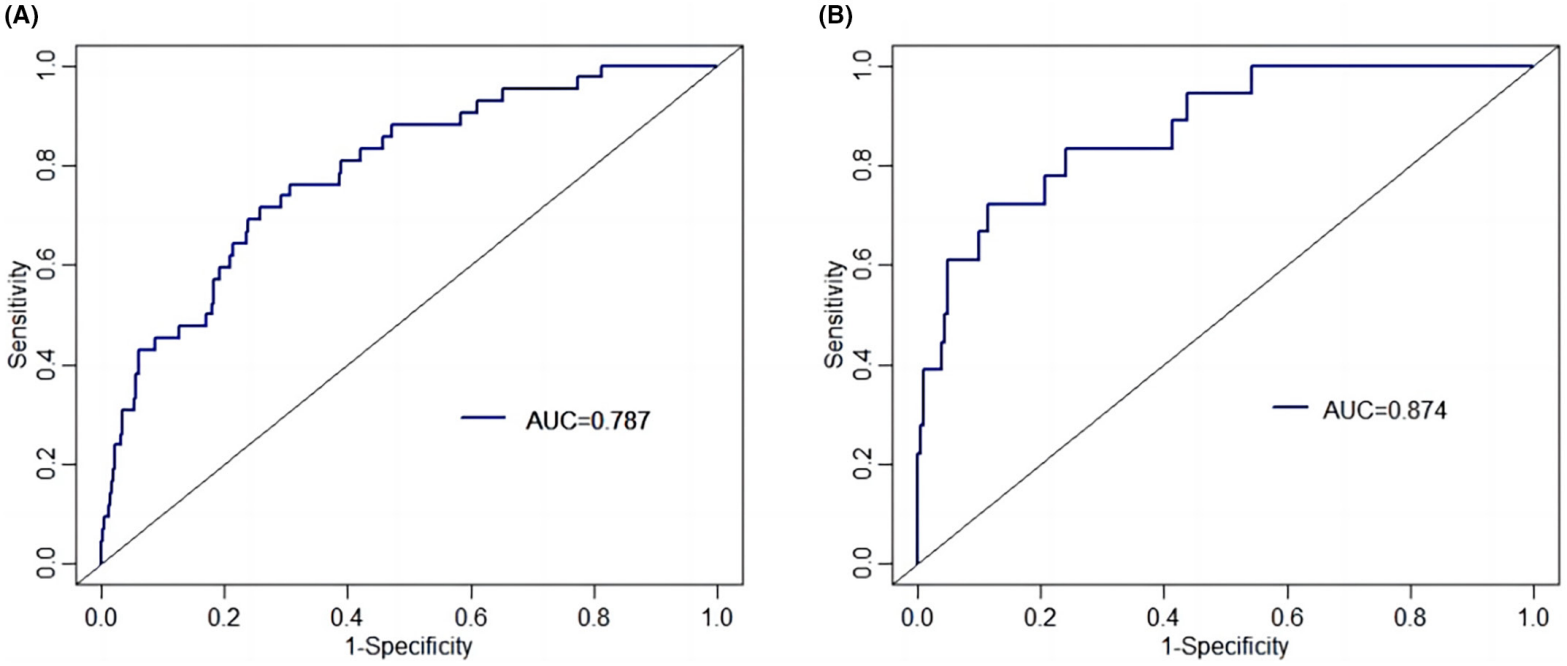
Evaluation curves of the nomogram model. (A) receiver operating characteristic (ROC) curve of training set; (B) ROC curve of testing set.

**FIGURE 4:**
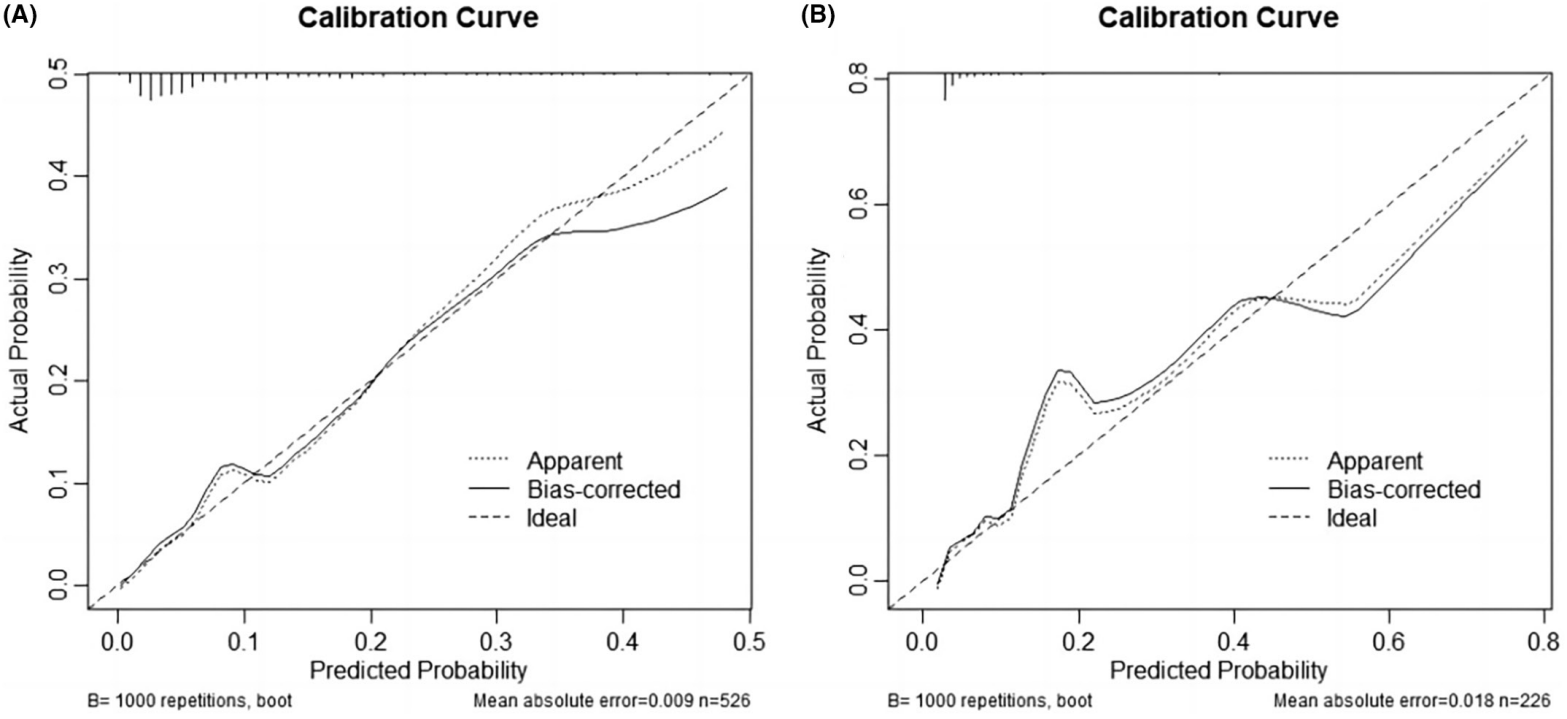
Evaluation curves of the nomogram model. (A) Calibration curve in training set; (B) calibration curves in testing set.

**FIGURE 5:**
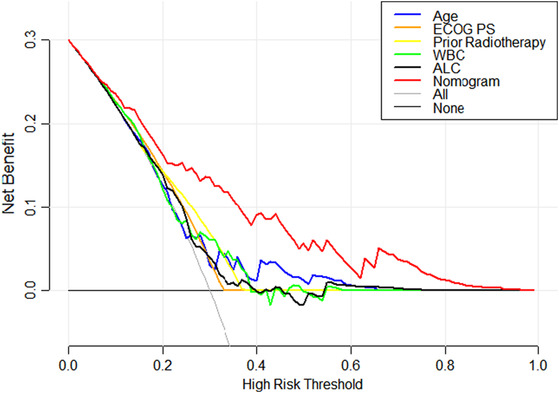
Decision curve analysis curves for the nomogram model in training set.

**FIGURE 6:**
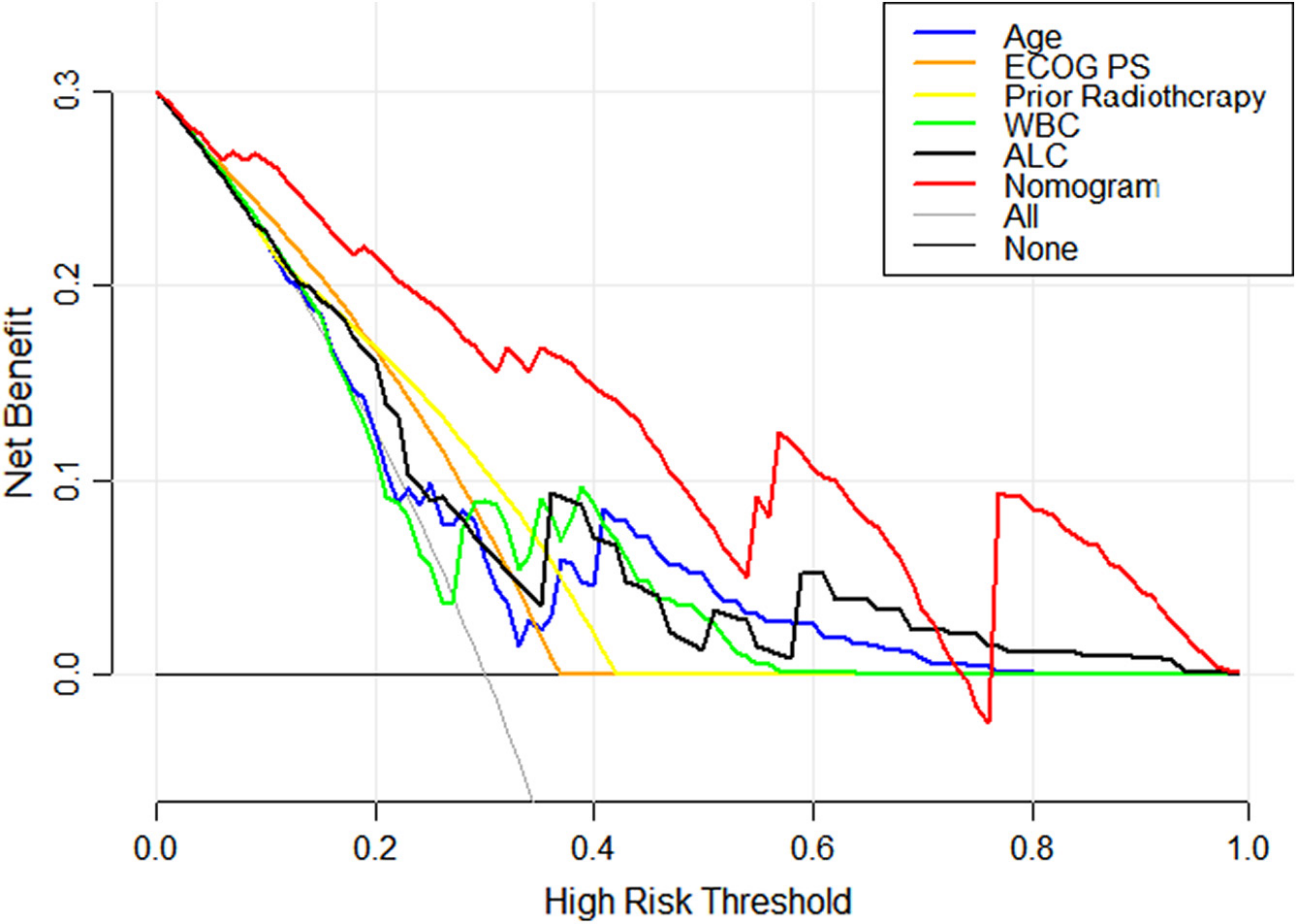
Decision curve analysis curves for the nomogram model in testing set.

The C‐index and the area under the ROC curve (AUC) were both 0.787 (95% CI: 0.716–0.857) and 0.874 (95% CI: 0.792–0.957) in the training set and testing set, respectively, indicating that the model has a better degree of discrimination (Figure 3). Meanwhile, the nomogram was validated by the 1000 repetitions of bootstrap sample corrections, and the mean absolute error was 0.009 in training set and 0.018 in testing set. The calibration curve of the nomogram shows how closely the predicted probability agreed with actual probability, which indicates the nomogram showed a powerful predictive ability of CIP (Figure 4). This study further evaluated the model's value for clinical application using DCA curves. The results show that the nomogram model could predict the risk of CIP occurrence in the training set better than other single prediction models in the entire range of risk thresholds (Figure 5). Likewise, the nomogram model in the testing set also showed good clinical utility, except for a slightly worse range of 0.74–0.76 thresholds (Figure 6).

### 3.4 Summary of clinical characteristics, treatment, and follow‐up information of all patients diagnosed with CIP

The information of all patients diagnosed with CIP is summarized in Table 4. Of the 752 patients included in this study, 60 were diagnosed with CIP (60/752, 8.0%), most of whom had grade 1–2 pneumonitis. In total population, grade 1–2 pneumonitis incidence was 4.7% (35/752) and 3.3% (25/752) for grade 3–4 pneumonitis, and most patients manifested with chest tightness, dyspnea, and cough. The median time from the beginning of immunotherapy to the onset of CIP was 106.5 days, the median number of immunotherapy cycles received was four. Of the 26 patients with a history of prior radiotherapy, 21 received thoracic radiotherapy (21/26, 80.8%). Regarding treatment, 85.0% of patients received glucocorticoids, and 11.7% of CIP patients received glucocorticoids combined with immunosuppressants. However, despite aggressive treatment, four patients died.

**TABLE 4:** Summary information of all patients diagnosed with CIP.

Variables	All (*N* = 60)
	*n* (%)
Grade	
G1	10 (16.7)
G2	25 (41.7)
G3–G4	25 (41.7)
Symptoms	
Asymptomatic	10 (16.7)
Cough	21 (35.0)
Chest tightness	48 (80.0)
Dyspnea	38 (63.3)
Fatigue	32 (53.3)
Fever	4 (6.7)
Cycles of immunotherapy, *n*	
Median (range)	4 (1–23)
Time from immunotherapy starting to onset of pneumonitis, days	
Median (range)	106.5 (7.0–557.0)
Prior radiotherapy	
Thoracic radiotherapy	21 (35.0)
Non‐thoracic radiotherapy	5 (8.3)
Treatment	
Glucocorticoids	51 (85.0)
Glucocorticoids combined with immunosuppressants	7 (11.7)
Restarting immunotherapy	
Yes	23 (38.3)
No	37 (61.7)

Abbreviation: CIP, checkpoint inhibitor‐related pneumonitis.

**FIGURE 7:**
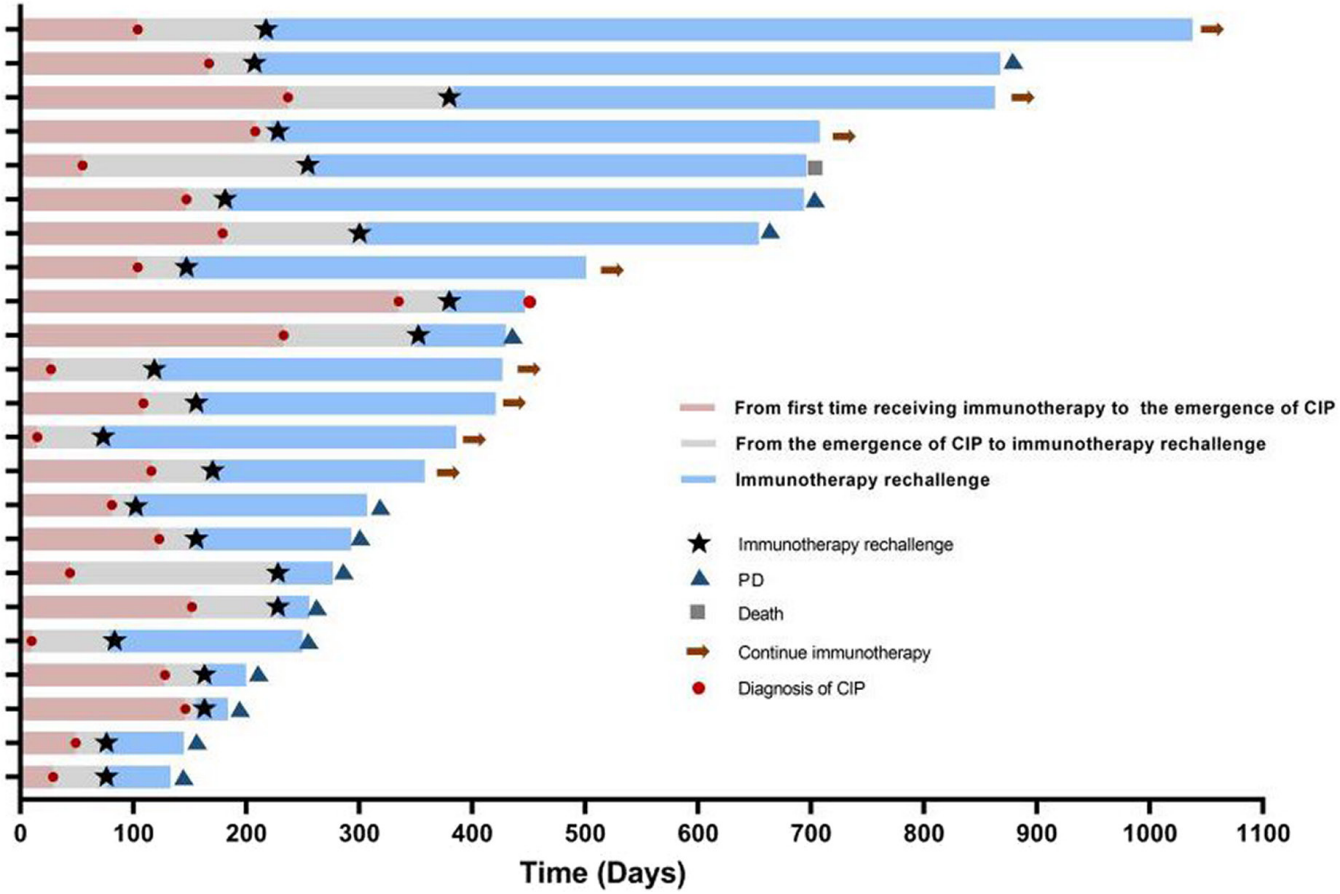
Outcomes in patients who started immunotherapy rechallenge.

Immunotherapy was restarted in 23 (38.3%) patients after treatment. The median time from the onset of CIP to the restart of immunotherapy was 51 days (range: 10–200 days). One patient developed CIP again at 70 days after the restart of immunotherapy and discontinued the ICI. Another patient died at 441 days after the immunotherapy restart due to the reoccurrence of CIP. As of July 30, 2022, 13 patients have experienced disease progression, and eight patients have stable disease and continue receiving immunotherapy (Figure 7).

## 4 DISCUSSION

This retrospective study analyzed 1476 advanced NSCLC patients receiving immunotherapy, ultimately including 752 patients based on stringent inclusion criteria. In the total population, 60 patients were diagnosed with CIP, and the incidence of all‐grade CIP was 8.0% (60/752), grade 1–2 pneumonitis was 4.7%, and grade 3–4 pneumonitis was 3.3%. The study identified five variables significantly associated with the occurrence of CIP by multivariate logistic analysis, which included age (>61 years), ECOG PS score of 2, higher baseline WBC (>7.1 × 10^9^/L), lower baseline ALC (<1.0 × 10^9^/L), and history of prior radiotherapy. Then, we use these five variables to develop a nomogram model to predict the occurrence of CIP. Further validation of the model revealed that it has good predictive accuracy, discrimination, and clinical utility to assist clinicians in predicting the risk of CIP.

Immunotherapy has innovatively widened the scope of lung cancer therapy and has become one of the standard treatments for advanced NSCLC in recent years.^17^ Frequently used ICIs (such as PD‐1 and PD‐L1 inhibitors) induce anticancer immunity by inhibiting the effect of immune checkpoint molecules and activating the effector T cells. However, excessive activation of the immunity may cause multi‐organ system irAEs.^18^ Previous research revealed that CIP is a relatively rare irAE in lung cancer immunotherapy but have potentially lethal.^9^ In clinical practice, some NSCLC patients with CIP continue to experience progressive respiratory function decline despite aggressive treatment, leading to respiratory failure and even fatality at a rate of 27%.^10^ Thus, it is crucial to identify the risk factors for CIP occurrence for ICIs toxicity management.

As the number of older individuals receiving ICI treatment continues to rise, it becomes increasingly important to understand the potential risk of irAEs for this specific subgroup. A retrospective real‐world study indicated that the elderly population (≥70 years) who received anti‐PD‐1/PD‐L1 therapy appears to be more susceptible to the development of immune‐related skin toxicity (35.0% vs. 11.3%, *p* = 0.047).^19^ Another multicenter retrospective study also found that elderly patients are up to twice as likely to discontinue immunotherapy due to irAEs as younger patients (30.9% vs. 15.1%, *p* = 0.008).^20^ The increased risk of pneumonitis in elderly patients treated with ICIs is also related to their physical condition at baseline. Patients with poorer ECOG PS are generally in an immunosuppressed state and prone to suffer CIP. Barrón et al.^11^ demonstrated that baseline ECOG PS was significantly higher in CIP patients than in non‐CIP patients (*p* < 0.01), which is consistent with our study. In our study, elder patients (*p* = 0.014, OR = 1.056, 95% CI: 1.011–1.102) and patients with poorer ECOG PS (*p* = 0.002, OR = 6.170, 95% CI: 1.943–19.590) are more likely to develop CIP. Even so, immunotherapy is more tolerable than chemotherapy for older people. The dose of ICI should be adjusted in elderly patients based on disease severity and physical condition.

Previous study indicated that prior radiotherapy history is associated with CIP for lung cancer patients receiving ICIs treatment.^11^ Radiotherapy could promote the capacity of tumor‐related T cells to identify and eliminate cancer cells through enhanced immune activity.^21^, ^22^ However, excessive immune activation may lead to numerous irAEs. Barrón et al.^11^ reported that among the 22 patients diagnosed with CIP in their study, 73% (16/22) had prior radiotherapy history (*p* < 0.001, OR = 6.04, 95% CI 2.03–18.0). Secondary analysis of the KEYNOTE‐001 trial revealed that patients with previous thoracic radiotherapy were also predisposed to pulmonary toxicity after pembrolizumab treatment (63% vs. 40%, *p* = 0.052).^23^ In our study, history of prior radiotherapy proves to be the most important predictor (*p* < 0.001; OR = 4.005; 95% CI = 1.920–8.355), providing further evidence for the effect of radiotherapy in CIP development. Contradictively, several clinical trials have suggested that previous radiotherapy may enhance the efficacy of immunotherapy. In PEMBRO‐RT Phase 2 trial, ORR at 12 weeks was 36% in the experimental arm (SBRT and pembrolizumab) versus 18% in the control arm (pembrolizumab without SBRT) (*p* = 0.07).^24^ In the secondary analysis of KEYNOTE‐001 trial, progression‐free survival (4.4 vs. 2.1 months, *p* = 0.019, HR = 0.56) and overall survival (10.7 vs. 5.3 months, *p* = 0.026, HR = 0.58) are significantly longer in patients who received any radiotherapy prior to immunotherapy than in patients without previous radiotherapy.^23^ Hence, whether to continue immunotherapy in patients who have received radiation therapy is still debatable.

The biological mechanism of CIP is not fully understood yet. Several studies have demonstrated that amplified pulmonary inflammation response may account for the occurrence of CIP.^4^, ^13^, ^25^ Lin et al.^12^ study revealed that elevated IL‐6 and high levels of IL‐10 correlates with the occurrence of CIP. Another study showed that lymphocytosis increased in bronchoalveolar lavage of patients with CIP, predominantly CD4^+^ T cells.^26^ Meanwhile, the CD4^+^ T cells can produce cytokines (such as IL‐4, IL‐5, IL‐6, IL‐9, IL‐10, and IL‐13) that ultimately leads to excessive inflammation response. These findings have demonstrated the dominating role of overly activated T lymphocytes in inflammatory lung damage and the pathogenesis of CIP. Similarly, we observed that higher baseline peripheral blood WBC (*p* < 0.001, OR = 1.604, 95% CI = 1.250–2.059) is significantly associated with CIP. Besides, our research also shows that the lower baseline peripheral blood ALC (*p* = 0.034; OR = 0.288; 95% CI = 0.091–0.909) was an independent biomarker for the occurrence of CIP. Lin et al.^12^ analyzed peripheral blood parameters in lung cancer patients and founded that reduced ALC levels were associated with the occurrence of grade 3–4 CIP (OR = 0.19, 95% CI = 0.03–1.08, *p* = 0.06). Decrease of ALC in peripheral blood may be caused by increased infiltration of lymphocytes into the site of inflammation. Another research also discovered that decreased ALC is an independent risk factor for irAEs (OR = 5.01, *p* = 0.012).^27^ In addition, lymphocytopenia has been proved to be negatively associated with the efficacy of ICI in NSCLC or other malignant cancer patients.^28^, ^29^ A retrospective study has demonstrated that low baseline ALC is an independent risk predictor of shortened PFS in NSCLC patients who receive immunotherapy.^28^ Ku et al. revealed that baseline lymphocytopenia (ALC <1000/uL) correlates with significantly worse clinical benefit outcomes (0% vs. 51%; *p* = 0.01) and OS (1.4 vs. 11.9 months; *p* < 0.001) in advanced melanoma patients receiving immunotherapy.^29^ These findings have clearly elucidated the importance of peripheral biomarker for predicting treatment response and adverse events in ICI‐treated populations.

In addition to the risk factors described above, pre‐existing interstitial lung disease and emphysema may also be associated with CIP. Still, there is insufficient evidence to confirm the association between these suspected factors and CIP. In a retrospective study of 54 patients with CIP, the researchers did not find a correlation between baseline pulmonary disease and CIP.^13^ Our study also did not observe a significant difference in the pre‐existing interstitial lung disease and emphysema between CIP and non‐CIP groups, but the incidence of patients in the CIP group was higher than in the non‐CIP group. The median time from immunotherapy to the onset of CIP in this study was 106.5 days. The research reports that the median time to onset of CIP is generally around 3 months, which is consistent with our study.^4^ There are no consistent recommendations regarding when to restart ICIs therapy after grade 1–2 CIP becomes alleviating.^16^ The immunotherapy response status of the tumor is an essential factor in determining whether to restart immunotherapy. In an observational study conducted by Dolladille, patients who developed CIP after receiving immunotherapy underwent immune rechallenge therapy in 21% of cases, but 27.7% of patients developed CIP again after restarting immunotherapy.^30^ In our study, immunotherapy was restarted in 23 (38.3%) patients after treatment. The median time from the onset of CIP to the restart of immunotherapy was 51 days (range: 10–200 days). One patient developed immune‐associated pneumonia again at 70 days after the restart of immunotherapy and discontinued the ICI. Another patient died at 441 days after the immunotherapy restart due to the reoccurrence of CIP. Therefore, the choice of immune rechallenge must be considered carefully to avoid the recurrence of more severe adverse events.

The main limitation of our research is that this study is a single‐center retrospective study, which could result in selection bias. A broader treatment population must be examined to confirm our findings. Furthermore, only internal and external validation of our institution's data was conducted in our study. Additional subcenters are required to validate our findings externally. In the future, we aim to conduct a multicenter prospective study to confirm our results.

## 5 CONCLUSION

Our study established a nomogram model for predicting the risk of CIP, which has good clinical discrimination and prediction accuracy, and is a practical clinical tool.

## AUTHOR CONTRIBUTIONS

**Yao Zhang:** Conceptualization (equal); data curation (equal); formal analysis (equal); investigation (equal); methodology (equal); project administration (equal); writing – original draft (equal). **Lincheng Zhang:** Conceptualization (equal); data curation (equal); formal analysis (equal); methodology (equal); writing – original draft (equal); writing – review and editing (equal). **Shuhui Cao:** Data curation (supporting); formal analysis (supporting); methodology (supporting); resources (supporting); software (supporting). **Yue Wang:** Conceptualization (supporting); data curation (supporting); validation (supporting); visualization (supporting). **Xuxinyi Ling:** Formal analysis (supporting); investigation (supporting); methodology (supporting); resources (supporting); software (supporting). **Yan Zhou:** Funding acquisition (lead); investigation (lead); methodology (lead); validation (lead); visualization (lead). **Hua Zhong:** Funding acquisition (lead); methodology (lead); project administration (lead).

## CONFLICT OF INTEREST STATEMENT

The authors declare no conflicts of interest.

## ETHICS STATEMENT

This retrospective study was approved by the institutional review board and ethics committee of Shanghai Chest Hospital (No. KS(Y)21310). The requirement for participants' informed consent was waived.

## Data Availability

The data that support the findings of this study are available from the corresponding author upon reasonable request.

## References

[1] Thai AA , Solomon BJ , Sequist LV , Gainor JF , Heist RS . Lung cancer. Lancet. 2021;398:535‐554. 34273294 10.1016/S0140-6736(21)00312-3

[2] Wang L , Yang Y , Yu J , et al. Efficacy and safety of anti‐PD‐1/PD‐L1 in combination with chemotherapy or not as first‐line treatment for advanced non‐small cell lung cancer: a systematic review and network meta‐analysis. Thorac Cancer. 2022;13:322‐337. 34907661 10.1111/1759-7714.14244 PMC8807232

[3] Shankar B , Zhang J , Naqash AR , et al. Multisystem immune‐related adverse events associated with immune checkpoint inhibitors for treatment of non‐small cell lung cancer. JAMA Oncol. 2020;6:1952‐1956. 33119034 10.1001/jamaoncol.2020.5012 PMC7596677

[4] Yin J , Wu Y , Yang X , Gan L , Xue J . Checkpoint inhibitor pneumonitis induced by anti‐PD‐1/PD‐L1 therapy in non‐small‐cell lung cancer: occurrence and mechanism. Front Immunol. 2022;13:830631. 35464480 10.3389/fimmu.2022.830631 PMC9021596

[5] Suresh K , Naidoo J , Lin CT , Danoff S . Immune checkpoint immunotherapy for non‐small cell lung cancer: benefits and pulmonary toxicities. Chest. 2018;154:1416‐1423. 30189190 10.1016/j.chest.2018.08.1048 PMC6335259

[6] Zhang Q , Tang L , Zhou Y , He W , Li W . Immune checkpoint inhibitor‐associated pneumonitis in non‐small cell lung cancer: current understanding in characteristics, diagnosis, and management. Front Immunol. 2021;12:663986. 34122422 10.3389/fimmu.2021.663986 PMC8195248

[7] Cho JY , Kim J , Lee JS , et al. Characteristics, incidence, and risk factors of immune checkpoint inhibitor‐related pneumonitis in patients with non‐small cell lung cancer. Lung Cancer. 2018;125:150‐156. 30429014 10.1016/j.lungcan.2018.09.015

[8] Brahmer JR , Lacchetti C , Schneider BJ , et al. Management of immune‐related adverse events in patients treated with immune checkpoint inhibitor therapy: American Society of Clinical Oncology Clinical Practice Guideline. J Clin Oncol. 2018;36:1714‐1768. 29442540 10.1200/JCO.2017.77.6385 PMC6481621

[9] Rashdan S , Minna JD , Gerber DE . Diagnosis and management of pulmonary toxicity associated with cancer immunotherapy. Lancet Respir Med. 2018;6:472‐478. 29856320 10.1016/S2213-2600(18)30172-3 PMC7341891

[10] Atchley WT , Alvarez C , Saxena‐Beem S , et al. Immune checkpoint inhibitor‐related pneumonitis in lung cancer: real‐world incidence, risk factors, and management practices across six health care centers in North Carolina. Chest. 2021;160:731‐742. 33621599 10.1016/j.chest.2021.02.032 PMC8411447

[11] Barrón F , Sánchez R , Arroyo‐Hernández M , et al. Risk of developing checkpoint immune pneumonitis and its effect on overall survival in non‐small cell lung cancer patients previously treated with radiotherapy. Front Oncol. 2020;10:570233. 33117699 10.3389/fonc.2020.570233 PMC7550759

[12] Lin X , Deng H , Yang Y , et al. Peripheral blood biomarkers for early diagnosis, severity, and prognosis of checkpoint inhibitor‐related pneumonitis in patients with lung cancer. Front Oncol. 2021;11:698832. 34327140 10.3389/fonc.2021.698832 PMC8313853

[13] Chu X , Zhao J , Zhou J , et al. Association of baseline peripheral‐blood eosinophil count with immune checkpoint inhibitor‐related pneumonitis and clinical outcomes in patients with non‐small cell lung cancer receiving immune checkpoint inhibitors. Lung Cancer. 2020;150:76‐82. 33080551 10.1016/j.lungcan.2020.08.015

[14] Chai R , Fan Y , Zhao J , He F , Li J , Han Y . Prognostic nomogram on clinicopathologic features and serum indicators for advanced non‐small cell lung cancer patients treated with anti‐PD‐1 inhibitors. Ann Transl Med. 2020;8:1078. 33145297 10.21037/atm-20-4297 PMC7575979

[15] Huang H , Chen Y , Weng X , Li S , Zhang L , Chen P . Development and validation of a nomogram for evaluating the prognosis of immunotherapy plus antiangiogenic therapy in non‐small cell lung cancer. Cancer Cell Int. 2022;22:261. 35989349 10.1186/s12935-022-02675-y PMC9394085

[16] Thompson JA , Schneider BJ , Brahmer J , et al. Management of immunotherapy‐related toxicities, version 1.2019. J Natl Compr Canc Netw. 2019;17:255‐289. 30865922 10.6004/jnccn.2019.0013

[17] Mamdani H , Matosevic S , Khalid AB , et al. Immunotherapy in lung cancer: current landscape and future directions. Front Immunol. 2022;13:823618. 35222404 10.3389/fimmu.2022.823618 PMC8864096

[18] Ramos‐Casals M , Brahmer JR , Callahan MK , et al. Immune‐related adverse events of checkpoint inhibitors. Nat Rev Dis Primers. 2020;6:38. 32382051 10.1038/s41572-020-0160-6 PMC9728094

[19] Paderi A , Fancelli S , Caliman E , et al. Safety of immune checkpoint inhibitors in elderly patients: an observational study. Curr Oncol. 2021;28:3259‐3267. 34449588 10.3390/curroncol28050283 PMC8395507

[20] Nebhan CA , Cortellini A , Ma W , et al. Clinical outcomes and toxic effects of single‐agent immune checkpoint inhibitors among patients aged 80 years or older with cancer: a multicenter international cohort study. JAMA Oncol. 2021;7:1856‐1861. 34734989 10.1001/jamaoncol.2021.4960 PMC8569601

[21] Martinov T , Fife BT . Fractionated radiotherapy combined with PD‐1 pathway blockade promotes CD8 T cell‐mediated tumor clearance for the treatment of advanced malignancies. Ann Transl Med. 2016;4:82. 27004229 10.3978/j.issn.2305-5839.2016.01.13 PMC4779769

[22] Sharabi AB , Lim M , DeWeese TL , Drake CG . Radiation and checkpoint blockade immunotherapy: radiosensitisation and potential mechanisms of synergy. Lancet Oncol. 2015;16:e498‐e509. 26433823 10.1016/S1470-2045(15)00007-8

[23] Shaverdian N , Lisberg AE , Bornazyan K , et al. Previous radiotherapy and the clinical activity and toxicity of pembrolizumab in the treatment of non‐small‐cell lung cancer: a secondary analysis of the KEYNOTE‐001 phase 1 trial. Lancet Oncol. 2017;18:895‐903. 28551359 10.1016/S1470-2045(17)30380-7 PMC5538772

[24] Theelen WSME , Peulen HMU , Lalezari F , et al. Effect of pembrolizumab after stereotactic body radiotherapy vs pembrolizumab alone on tumor response in patients with advanced non‐small cell lung cancer: results of the PEMBRO‐RT phase 2 randomized clinical trial. JAMA Oncol. 2019;5:1276‐1282. 31294749 10.1001/jamaoncol.2019.1478 PMC6624814

[25] Naidoo J , Wang X , Woo KM , et al. Pneumonitis in patients treated with anti‐programmed death‐1/programmed death ligand 1 therapy. J Clin Oncol. 2017;35:709‐717. 27646942 10.1200/JCO.2016.68.2005 PMC5559901

[26] Suresh K , Naidoo J , Zhong Q , et al. The alveolar immune cell landscape is dysregulated in checkpoint inhibitor pneumonitis. J Clin Invest. 2019;129:4305‐4315. 31310589 10.1172/JCI128654 PMC6763233

[27] Fujisawa Y , Yoshino K , Otsuka A , et al. Fluctuations in routine blood count might signal severe immune‐related adverse events in melanoma patients treated with nivolumab. J Dermatol Sci. 2017;88:225‐231. 28736218 10.1016/j.jdermsci.2017.07.007

[28] Pike LRG , Bang A , Mahal BA , et al. The impact of radiation therapy on lymphocyte count and survival in metastatic cancer patients receiving PD‐1 immune checkpoint inhibitors. Int J Radiat Oncol Biol Phys. 2019;103:142‐151. 30227198 10.1016/j.ijrobp.2018.09.010

[29] Ku GY , Yuan J , Page DB , et al. Single‐institution experience with ipilimumab in advanced melanoma patients in the compassionate use setting: lymphocyte count after 2 doses correlates with survival. Cancer. 2010;116:1767‐1775. 20143434 10.1002/cncr.24951 PMC2917065

[30] Dolladille C , Ederhy S , Sassier M , et al. Immune checkpoint inhibitor rechallenge after immune‐related adverse events in patients with cancer. JAMA Oncol. 2020;6:865‐871. 32297899 10.1001/jamaoncol.2020.0726 PMC7163782

